# How do futsal players of different categories play during official matches? A tactical approach to players’ organization on the court from positional data

**DOI:** 10.1371/journal.pone.0199619

**Published:** 2018-06-26

**Authors:** Murilo José de Oliveira Bueno, Fabio Giuliano Caetano, Michelle Kaori Yonezawa, André Santana Grella, Sergio Augusto Cunha, Felipe Arruda Moura

**Affiliations:** 1 Sport Sciences Department, State University of Londrina, Londrina, Brazil; 2 College of Physical Education, University of Campinas, Campinas, Brazil; Sao Paulo State University - UNESP, BRAZIL

## Abstract

The purpose of this study was to analyze futsal players’ organization on the court in different categories while attacking and defending, in interception and shot to goal situations. We obtained the trajectories of 89 players from the under-15 category, 102 players from the under-18 category, and 110 professional players, during official matches. The spread, surface area, and Euclidian distances between the teams’ centroids were measured to represent the distribution of the futsal players on the court. The variables were analyzed during each offensive and defensive sequence, and during situations of shots to goal and interceptions, with and without the outfield goalkeeper player participation. While the players were attacking, all categories presented greater spread and surface area, compared to values when players were defending (*P* < 0.01). Among the categories, the results showed lower spread and area values for the younger players (*P* < 0.01). The results of spread, surface area, and distances between the teams’ centroids showed different forms of organization for each of the categories in specific situations of shots to goal and interceptions. The study provided insights that allow coaches to better plan suitable tactical training according to the requirements of each category.

## Introduction

Due to technological advances, a significant amount of research has been performed to provide detailed information about team sports matches from players positional data and to provide information for training enhancement and evaluation of teams during official matches [1–5]. In a tactical context, studies have sought to understand the behavior of the dynamics of interpersonal coordination between attacking players and supporters/defenders during offensive sequences of futsal matches [6–8]. In addition, variables such as the team stretch index, defined as the average of the players’ distances from the geometric center of the team [9], the surface area, defined as the total space occupied by the team, and the spread, defined as a measure of distance between players from the same team as a function of time, have been analyzed in team sports to describe players organization on the pitch [1,4].

In specific situations of a match, team organization can change as a result of a perturbation, such as loss of ball possession or scoring a goal [1]. An analysis of the offensive sequences resulting in goals showed that sequences started by a fast transition from defense to attack (i.e., counter-attack) are one of the most frequent [10], so team organization in both phases of the match can be decisive. From data on the surface area and distance between the centroid of futsal teams during an international friendly match, a recent study characterized the organization of the players on the court during situations of interceptions and shots to goal [3]. The results showed that when the teams performed interceptions, they presented larger area values, compared to situations in which they suffered shots to goal. In addition, the distances between the centroids were smaller when interceptions were performed, suggesting that the greater the proximity between teams, the greater the chances of success in defense.

Although these analyses have brought important contributions to the understanding of futsal dynamics, they may represent only the characteristics of professional teams. It is possible to verify that youth practitioners have difficulty understanding how to organize themselves on the court according to the position of their teammates and opponents. Thus, youth practitioners may present a distribution on the court that is completely different from a professional match.

The collective behavior for different small-sided soccer games, represented by a dispersion index (ratio between the maximum distance value among teammate players in the longitudinal and lateral axis of the court) and by the distance between the teams’ centroids in three different age groups (under-9, under-11, and under-13) was presented in the literature [11]. Folgado et al. [11] reported that younger players tend to participate in the game only when they are closer to the ball, presenting individual behavior and participating less in collective strategies in comparison to older players. In another recent study [12], the time series of the surface area, dispersion index of the players, and lateral and longitudinal displacement of the teams on the pitch in three different categories (under-16, under-17, and under-19) in small-sided games were reported. Barnabe et al. [12] found greater surface area values for older players when compared to younger players in attacking situations. Additionally, the older players presented more stable collective behavior and more effective tactical organization. These studies showed that the effects of age or experience influence the tactical behavior of teams. Both studies reported that older players (and possibly more experienced) present more collective behavior, concerning the dispersion and displacement of players in attack and defense contexts. However, these effects are still unknown for players from different categories during official futsal matches.

From data about players’ position as a function of time, from youth levels to professional, it is possible to describe the tactical organization on the court of teams in different categories. Thus, by understanding the tactical transition from young level stages to the professional stage, as well as the difficulties that they may find during training programs, it is possible to provide better conditions to coaches to plan and develop appropriate exercise programs for each age group. This information about the characteristics of the game and teams of different categories is relevant for better understanding of the sport, since match analysis and players development are identified as lacking the literature of futsal [13].

Therefore, the aim of this study was to characterize futsal teams’ organization on the court, from different categories, during attacking-defending contexts and during situations of interceptions and shots to goal. Specifically, we were interested in examining whether teams from different categories have different organization on the court when they are with or without the ball. For specific situations of interceptions and shots to goal, we evaluated whether the teams presented different organization on the court when they successfully performed defensive and offensive actions, compared to unsuccessful actions.

## Materials and methods

### Data collection

The Ethics Committee of the State University of Londrina approved this study (process number: 22514). Five official matches were filmed in each of the three categories analyzed: under-15 (U15), under-18 (U18), and professional (PRO), at three different championship levels (regional, state, and national league, respectively), resulting in 15 matches from 30 different teams. Three digital cameras (30 Hz) were fixed at elevated positions in the gymnasiums. The study tracked the trajectories of 301 players (n = 89 for U15 players, n = 102 for U18 players, and n = 110 for PRO players). The trajectory of each player was obtained using an automated tracking system via DVideo software [14,15]. The average error for the determination of player position was 0.098 m, and the average error for the distance covered was 0.8% [16].

Each player from each team was numbered as p = 1, 2, …, 14. Thus, the two-dimensional coordinates of the players are defined as *p*(*Xp* (*t*), *Yp* (*t*)), where *t* represents each instant of time (in seconds). Finally, players’ trajectories were filtered with a Butterworth third-order low-pass digital filter with a cut-off frequency of 0.4 Hz. With the smoothed trajectories of all players, we calculated teams’ spread and surface area as a function of time. Additionally, we identified, in each instant of time, which team had possession of the ball, as well as the interceptions and shots to goal performed according to the criteria defined by Moura et al. [5].

For each instant of time *t*, we calculated the Euclidean distances of each player and their teammates. The distances between players were organized in a symmetric matrix *D* of order *m x n*, where *m* = number of distance values between players of the same team and *n* = corresponding frames at each time *t*.

The Euclidean norm of each vector of the matrix *D* was then calculated, corresponding to spread values at each time *t*, according to the equation:

$$\|D_{n}\|=\sqrt{\sum_{j=1}^{p}{\left|{d_{n}}_{ij}(t)\right|}^{2}}$$ (1)

Where *p* represents the number of players on the court of the same team and ${d_{n}}_{ij}$ represents the value of Euclidean distance between each pair of players from the same team. According to Moura et al. [4], larger ||D_n_|| values mean that players are more spread across the court. In contrast, lower values indicate the players present a more compact structure.

The surface area was represented by the convex hull area, calculated from the position of the players of the same team. The identification of vertices of the convex hull and the area were calculated at each instant of time *t* using the quickhull technique [17].

The values of spread and surface area were normalized by the maximum possible value that a team can present on the court [18], and thus presented as a percentage relative to the maximum possible value. This form of presentation was adopted as the matches were held in different locations, with variation in the dimensions of the courts, a fact that may interfere directly in the comparison of the variables. Finally, for each variable, we calculated the average values for each offensive and defensive sequence (sequences when team was with and without ball possession, respectively). The centroid of each team can be defined as the average of the 2D coordinates of the teammates [3,9]. Subsequently, the Euclidian distance between teams’ centroids was determined.

The spread, surface area, and distance between the centroids of the teams values were analyzed in specific situations of shots to goal and interceptions, when the teams were playing with or without participation of the outfield goalkeeper for the categories U15 (without outfield goalkeeper: n = 290 and 717, for shots to goal and interceptions, respectively; with outfield goalkeeper: n = 0, for both situations), U18 (without outfield goalkeeper: n = 312 and 748; with outfield goalkeeper: n = 40 and 27, for shots to goal and interceptions, respectively), and PRO (without outfield goalkeeper: n = 288 and 463; with outfield goalkeeper: n = 15 and 19, for shots to goal and interceptions, respectively). Teams centroids distance, spread, and surface area percentage values were identified in the exact frame in which the teams performed an interception or suffered a shot to goal.

### Statistical analysis

Before each analysis, a Levene’s variance test for homoscedasticity of data was applied. As no tests demonstrated homoscedasticity, a Box-Cox transformation was performed to reduce anomalies and heteroscedasticity of the values of spread and surface area in the conditions with and without the ball. Next, a two-way analysis of variance was performed to compare the spread and surface area percentage values in two factors: when the teams were with and without the ball (factor 1) and between categories (factor 2). When differences were found, a Tukey post-hoc test was applied to provide specific information on which data were significantly different from each other. The values are expressed as median and interquartile range. The effect size for the variance analysis was calculated according to Cohen’s *f* [19].

For analysis of the percentage values of spread, surface area, and distance between the centroids in situations of shots to goal and interceptions, primarily, a Lilliefors test was performed to check data normality. As the data did not present normal distribution, statistical inferences were made by non-parametric tests. A Wilcoxon rank-sun test was used to verify if the defending teams had different values when interceptions were performed compared to when the team suffered shots to goal. The same test was performed for situations when the teams were attacking, verifying if the values were different when teams performed shots to goal compared to when they suffered interceptions. For distances between the centroids, the Wilcoxon rank-sun test was used to verify whether there were differences when the teams performed interceptions compared to when they performed shots to goal. The analyses were performed separately for when the teams had or did not have participation of the outfield goalkeeper. For all analyses, we adopted a significance level of *P* < 0.05 and the effect size (*ES*) for the independent analysis was calculated and presented according to Cohen’s *d* [19].

## Results

Table 1 presents the spread and surface area percentage values when teams were with and without ball possession, for all categories. According to the statistical analysis, the teams presented greater spread values when they had ball possession, compared to the condition in which they did not have ball possession, for all categories (F = 6065.47; *P* < 0.01; *ES* = 0.62). Among the categories, the results showed lower values for the younger players (F = 9671.85; *P* < 0.01; *ES* = 0.59). The interaction among factors (F = 5711.32, *P* < 0.01; *ES* = 0.66) demonstrated that, in relation to the professional category, the younger the category was, the smaller the spread values when teams had ball possession. Without ball possession, the U15 category presented lower values compared to the U18 and PRO.

**Table 1:** Median (interquartile range) values of the percentage of spread and surface area for attack and defense sequences in relation to the maximum possible values for the categories during situations with and without the ball possession.

Categories	% Spread		% Surface area	
	With ball possession	Without ball possession	With ball possession	Without ball possession
**U15**	35.9 (9.0)*^#^	32.8 (15.8)^#^^†^	9.6 (5.1)*^#^^†^	6.4 (3.2)^†^
**U18**	38.5 (10.5)*^#^	35.1 (16.1)	11.9 (6.5)*^#^	7.1 (4.1)^#^
**PRO**	39.2 (9.2)*	33.6 (18.1)	12.2 (5.7)*	5.9 (3.5)

U15: Under-15; U18: Under-18; PRO: Professional.

*: Significantly different from without ball possession group.

^#^: Significantly different from the PRO category.

^†^: Significantly different from the U18 category.

For surface area, the values were higher while the teams had ball possession, compared to the condition when they were without the ball, for all categories (F = 407.69; *P* < 0.01; *ES* = 0.27). The results showed that the surface area values were different between the categories (F = 5117.0; *P* < 0.01; *ES* = 0.91). The interaction presented significant differences between the contexts related with ball possession among categories (F 360.77; *P* < 0.01; *ES* = 0.26). However, without ball possession, the U15 showed lower values compared to the U18. The U18 category presented greater values compared to the PRO. The *ES* represented large effects for almost all comparisons, except for the difference between categories for the surface area when the ball possession condition was considered.

**Fig 1:**
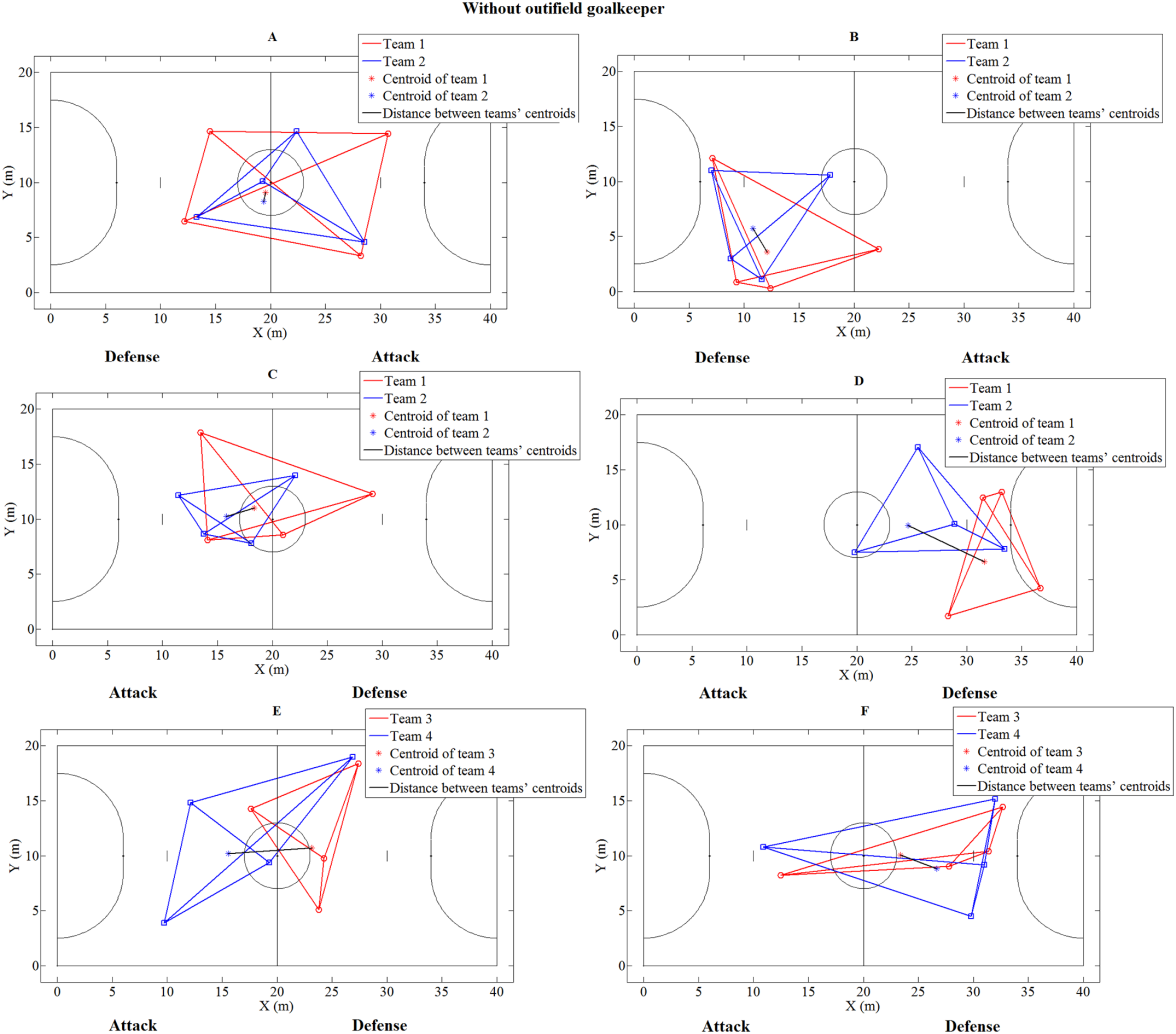
Players’ organization during interception and shots to goal situations without outfield goalkeeper. (A) U15: When team 1 performed an interception, (B) U15: When team 2 performed a shot to goal, (C) U18: When team 1 performed an interception, (D) U18: When team 2 performed a shot to goal, (E) PRO: When team 3 performed an interception, and (F) PRO: When team 4 performed a shot to goal.

Fig 1 shows examples of players organization on the court represented by team spread and surface area, for both teams, during specific situations of interceptions and shots to goal, without the participation of the outfield goalkeeper, for all the categories. A graphical exploratory analysis showed that U15 teams tend to present greater spread values (visually described by teammates distances) when performing interceptions, compared to situations when they suffered shots to goal. These results were confirmed in Table 2. For interception situations, without the participation of the outfield goalkeeper, the Wilcoxon test showed that only the U15 presented higher spread values when performing interceptions compared to when they suffered shots to goal.

**Table 2:** Median (interquartile range) spread values expressed in percentages in relation to the maximum possible values for categories without the participation of the outfield goalkeeper during defending and attacking situations, when interceptions or shots to goal occurred.

**Categories**	**Defending situation**		***P***	***ES***
	**When interceptions were performed**	**When teams suffered shots to goal**		
**U15**	30.0 (16.8)*	28.6 (19.4)	0.02	0.15
**U18**	32.0 (17.8)	31.1 (22.3)	0.33	0.06
**PRO**	31.6 (21.6)	32.0 (22.4)	0.98	< 0.01
	**Attacking situation**			
	**When shots to goal were performed**	**When teams suffered interceptions**		
**U15**	33.2 (10.3)	33.4 (10.2)	0.23	0.10
**U18**	37.1 (10.9)	37.0 (10.7)	0.86	0.02
**PRO**	37.9 (10.2)	38.5 (9.5)	0.84	0.05

U15: Under-15; U18: Under-18; PRO: Professional.

*: Significantly different from when suffered shots to goal group.

**Fig 2:**
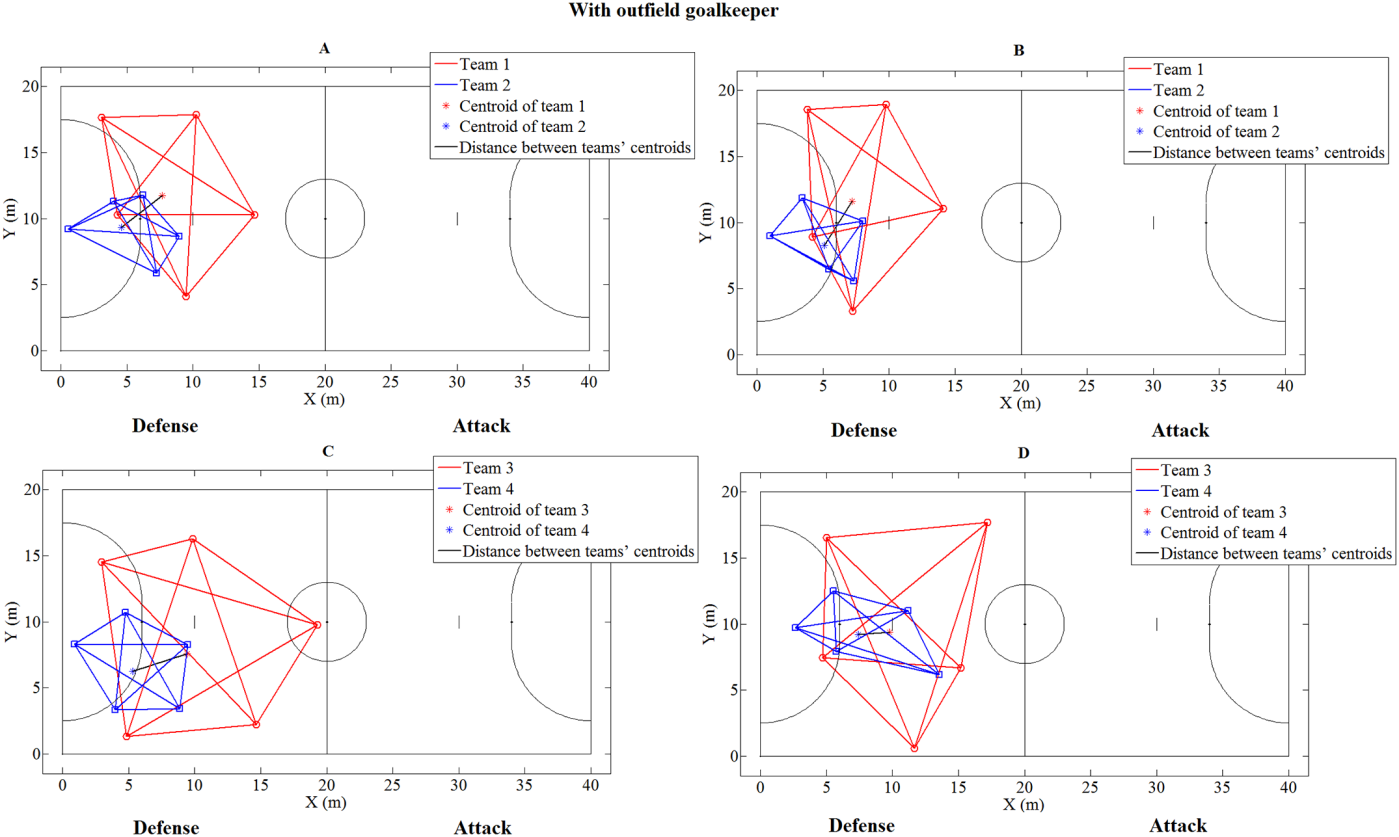
Players’ organization during interception and shots to goal with outfield goalkeeper. (A) U18: When team 2 performed an interception, (B) U18: When team 1 performed a shot to goal, (C) PRO: When team 4 performed an interception, and (D) PRO: When team 3 performed a shot to goal.

Fig 2 presents examples of surface area and spread for both teams during interceptions and shots to goal with the participation of the outfield goalkeeper for U18 and PRO. An exploratory analysis showed that the teams spread values, for both categories, were similar when they performed interceptions or suffered shots to goal. With the participation of the outfield goalkeeper, there were no significant differences between the conditions (both for defensive and offensive contexts), associated with success of interceptions or shots to goal (Table 3).

**Table 3:** Median (interquartile range) spread values expressed in percentages in relation to the maximum possible values for categories with the participation of the outfield goalkeeper during defending and attacking situations, when interceptions or shots to goal occurred.

**Categories**	**Defending situation**		***P***	***ES***
	**When interceptions were performed**	**When teams suffered shots to goal**		
**U15**	NA	NA	NA	NA
**U18**	35.5 (22.9)	34.4 (19.4)	0.37	0.27
**PRO**	33.0 (23.2)	33.3 (18.4)	0.67	0.10
	**Attacking situation**			
	**When shots to goal were performed**	**When teams suffered interceptions**		
**U15**	NA	NA	NA	NA
**U18**	38.4 (11.7)	36.6 (12.3)	0.74	0.06
**PRO**	38.6 (3.9)	38.8 (8.9)	0.13	0.11

U15: Under-15; U18: Under-18; PRO: Professional.

NA: Not applicable (there were no situations with the participation of the outfield goalkeeper).

With no participation of the outfield goalkeeper, the surface area results demonstrated that the U15 and U18 categories showed differences (*P* < 0.05) in the defensive condition (Table 4). The U15 and U18 presented higher values when they performed interceptions than when they suffered shots to goal.

**Table 4:** Median (interquartile range) surface area values expressed in percentages in relation to the maximum possible values for categories during defending and attacking situations without the participation of the outfield goalkeeper when interceptions or shots to goal occurred.

**Categories**	**Defending situation**		***P***	***ES***
	**When interceptions were performed**	**When teams suffered shots to goal**		
**U15**	4.9 (4.2)*	3.5 (3.1)	< 0.01	0.41
**U18**	4.9 (4.7)*	3.8 (3.6)	< 0.01	0.26
**PRO**	3.7 (3.6)	3.6 (3.4)	0.64	0.02
	**Attacking situation**			
	**When shots to goal were performed**	**When teams suffered interceptions**		
**U15**	9.1 (4.8) ^#^	8.2 (5.9)	< 0.01	0.20
**U18**	10.6 (6.9)	10.8 (6.8)	0.92	0.01
**PRO**	11.3 (8.0)	11.4 (7.5)	0.96	0.03

U15: Under-15; U18: Under-18; PRO: Professional.

*: Significantly different from when suffered shots to goal group.

^#^: Significantly different from when suffered interceptions group.

With the participation of the outfield goalkeeper, the U18 category teams showed lower surface area values (*P* < 0.05) when they performed interceptions than when they suffered shots to goal (Table 5).

**Table 5:** Median (interquartile range) surface area values expressed in percentages in relation to the maximum possible values for categories during defending and attacking situations with the participation of the goalkeeper when interceptions or shots to goal occurred.

**Categories**	**Defending situation**		***P***	***ES***
	**When interceptions were performed**	**When teams suffered shots to goal**		
**U15**	NA	NA	NA	NA
**U18**	3.5 (2.7)*	4.6 (5.7)	0.04	0.34
**PRO**	4.0 (2.6)	4.7 (3.4)	0.62	0.34
	**Attacking situation**			
	**When shots to goal were performed**	**When teams suffered interceptions**		
**U15**	NA	NA	NA	NA
**U18**	14.7 (9.7)	14.0 (7.4)	0.74	0.14
**PRO**	15.5 (8.7)	19.4 (6.8)	0.21	0.30

U15: Under-15; U18: Under-18; PRO: Professional.

NA: Not applicable (there were no situations with the participation of the outfield goalkeeper).

*: Significantly different from when suffered shots to goal group.

Finally, for the values of distance between the teams’ centroids with and without the outfield goalkeeper, the PRO category showed greater distances (*P* <0.05) when they performed interceptions than when they performed shots to goal (Table 6). In the specific analyses of shots to goal and interceptions, small and medium effects were found for almost all analyses, except for the U15 surface areas in defending situations and for the PRO centroid values with the outfield goalkeeper (large effects).

**Table 6:** Median (interquartile range) of distance (m) between the centroid of the teams during interceptions or shots to goal, with and without the participation of the outfield goalkeeper.

**Category**	**Without outfield goalkeeper**		***P***	***ES***
	**Interceptions**	**Shots to goal**		
**U15**	2.56 (2.08)	2.55 (1.93)	0.50	0.02
**U18**	3.13 (2.34)	3.08 (2.26)	0.97	0.03
**PRO**	3.69 (2.46)*	3.09 (2.46)	< 0.01	0.21
	**With outfield goalkeeper**			
	**Interceptions**	**Shots to goal**		
**U15**	NA	NA	NA	NA
**U18**	3.08 (1.97)	2.53 (2.31)	0.18	0.33
**PRO**	3.27 (2.42)*	1.36 (1.34)	0.01	0.91

U15: Under-15; U18: Under-18; PRO: Professional.

NA: Not applicable (there were no situations with the participation of the outfield goalkeeper).

*: Significantly different from shot to goal situation.

## Discussion

The purpose of this study was to analyze quantitatively the organization of futsal players on the court during official matches of different categories, when they had and did not have possession of the ball and in specific situations of interceptions and shots to goal. In general, when futsal teams had possession of the ball, greater spread and surface areas across the court were found, compared to when they did not have possession of the ball. The U15 category presented smaller spread compared to all categories. For the surface area, the U18 category presented greater values compared to the PRO category and the U15 category presented smaller surface area compared to the U18. Regarding players organization in specific situations of interceptions and shots to goal, the values of spread, surface area, and distance between the centroids of the teams demonstrated organization values when the teams made interceptions. The *ES* presents information about how certain phenomena may be present in a population [19], i.e., observing the results of the present study, the majority of the analyses presented a medium chance that the phenomenon could happen. However, it should be noted that in relation to the surface area, comparing the categories, this phenomenon may have a larger chance of occurrence, as well as for the distance values between the centroids with an outfield goalkeeper.

Ball possession exchanges between teams affect the distribution of players on the court during a match. Quantitative analysis of the distribution of players on the court can help to understand the dynamics of teams organization during attacking-defending contexts [4]. The results of this study showed that when a futsal team had possession of the ball, the values of distance between teammates and coverage area increased. Conversely, when the team was without ball possession, the players moved closer to each other and reduced the pitch coverage area, presenting more compact behavior and occupying a smaller area of the court. Similar behaviors have been reported in football [4,9]. When defending, some of the teams’ objectives were to avoid shots on their goal and recover ball possession. When attacking, the players organized themselves to increase shots on goal opportunities and to avoid being intercepted [20]. Thus, regardless of the category, it was found that athletes are able to organize themselves properly on the court, depending on the ball possession condition (i.e., whether they are attacking or defending).

The U15 surface area without ball possession presented no differences compared to PRO teams. Although these categories present similar strategies regarding coverage area when defending, the spread values showed that younger players (U15) presented more compact distribution, compared to U18 and PRO players. This outcome is understandable as team surface area and spread provide particular information about players’ distribution [4]. Therefore, contradictory results for spread and surface area may indicate that U15 and PRO, without ball possession, present similarities in space coverage but differences with respect to how teammates interact, represented by their respective distances (i.e., spread values).

When the players had possession of the ball, the U15 and U18 categories presented more compact distributions in comparison to the PRO. Generally, these results may indicate that younger levels have poor organization on the court and a possible inability to properly fill the spaces of the court available when they are attacking. These team behaviors suggest that younger players tend to solve the challenges of the match individually, approaching more of the ball and not performing collective behavior as a function of their teammates’ positions [11]. This information is valuable since it shows that U18 players, who should already present a high degree of sport learning, present limited collective behavior. However, this player tactical behavior may represent different game characteristics of each category and coaches should consider this outcome during their interventions for the development of players, mainly when players are close to category transition.

The spread and surface area variables, and distance between the centroids of the teams, analyzed in specific situations of interceptions and shots to goal, could provide important information about how teams are organized when they gain success or failure in defending and attacking actions [3,4]. When U15 teams were in a defensive condition, they were more spread across the court when performing interceptions than when suffering submissions. When observing the surface area results, the U15 teams also showed a larger area on the court when successfully completing defensive and offensive actions, both in situations without the participation of the outfield goalkeeper. These results suggest that U15 teams may have higher chances of performing an interception when they mark opposition players individually, spreading over the court and seeking to remain closer to their opponents (Fig 1). In attacking situations, larger surface area values can also indicate appropriate behavior to perform a shot to goal, allowing more space to perform passing sequences to teammates, receive the ball, dribble, and then perform a shot to goal.

For U18, without participation of the outfield goalkeeper, the area covered by the teams when they had success in performing interceptions where larger than when they suffered shots to goal. On the other hand, when the teams which involved the outfield goalkeeper achieved success in performing an interception, the area was smaller than when suffering shots to goal. These results are similar to those reported in the literature [3] for an international futsal match. Moura et al. [3] showed that when teams performed interceptions, the surface area was larger compared to situations when they suffered shots to goal. It is possible to argue that when teams play with the same number of players (i.e., none of them play with the outfield goalkeeper), individual marking, in which most players focus on marking a specific player during the match, can be effective for a successful interception. This behavior was also visualized when the pass performance was analyzed in situations without the outfield goalkeeper, with higher values of surface area of the defending team and smaller distance between opponent players being reported when unsuccessful passes of the attacking team occurred [21]. However, when the defensive team played with less players (i.e., when the attacking team worked with an outfield goalkeeper), the defensive team was more able to succeed in making an interception while occupying a smaller area and when it was closer to the goal (Fig 2). These findings corroborate the literature, which can hamper other opponent actions (for example, an opponent pass between the free spaces of the court) [22]. These actions may induce the opponent to make errors and consequently improve the chances of a successful interception, once as a previous study showed that in situations with participation of the outfield goalkeeper, a smaller surface area of the defensive team was related to unsuccessful passes of the attacking team [21].

Another variable that could explain the success and failure in situations of interception and shots to goal is the distance between the centroid of the teams, providing information about the ‘pressure’ that one team can have on the other [1,3]. Observing the professional category, when shots to goal occurred, the distances between the centroids were smaller than in situations in which interceptions were performed, regardless of the participation of the outfield goalkeeper. These results do not corroborate those found in the literature, which reported that futsal teams presented closer centroids when interceptions occurred, compared to situations in which shots to goal were performed [3]. However, the literature study does not consider situations involving the participation of the outfield goalkeeper. These are important results that could guide coaches to organize the teams appropriately on court to ensure better chances of success in performing interceptions or shots to goal.

Coaches should be alert to changes in behavior that may occur during a match in each category. The development of tactical training should be performed according to the characteristics of each category. According to the results presented, in younger categories, a defense may have greater success if players are trained to organize themselves in order to mark the opponents individually. However, for the professional category, remaining distant from the opponent may be more effective. Furthermore, coaches should be alert to the fact that, when players change categories, they need to be trained to adapt to the match demands required by the new category. Thus, the coach must deal with the different tactical demands faced by players when they change from one category to another and thus aid the tactical evolution of players throughout their careers in futsal.

For this study, a video-based system was used to acquire the position data of the players on the court. This method presents great precision and low-cost for its application, however it should be emphasized that it demands a great amount of time for the image processing, which limits data collection in real time with a large sample. Therefore, even though the effect size was controlled, generalization of the conclusions drawn could be limited to this sample. However, we emphasize that the results are of great value, considering that the study explored the organization of players during official matches.

## Conclusions

The results of this study showed that futsal teams in different categories organize themselves distinctly in official matches. It was possible to verify that players of younger categories play more compactly in relation to the professional category, which demonstrates the different behaviors and tactical demands in each category analyzed. In addition, different organizations on the court can determine the success in shots to goal or interception actions in each category. Experts could benefit from these results to understand how players of different categories organize themselves during official matches and devise specific training plans so that players can meet the specific tactical demands of play in each category, and to condition them for possible future transitions.
